# Overview of Innate Immune Cell Landscape in Liver Aging

**DOI:** 10.3390/ijms25010181

**Published:** 2023-12-22

**Authors:** Yan Lin, Qiao Li, Guangyu Liang, Nanyin Xiao, Jiankun Yang, Xiao Yang, Heng Zhang, Cuntai Zhang, Anding Liu

**Affiliations:** 1 Experimental Medicine Center, Tongji Hospital, Tongji Medical College, Huazhong University of Science and Technology, Wuhan 430030, China; 2 Key Laboratory of Vascular Aging, Ministry of Education, Tongji Hospital, Tongji Medical College, Huazhong University of Science and Technology, Wuhan 430030, China; 3 Department of Geriatrics, Tongji Hospital, Tongji Medical College, Huazhong University of Science and Technology, Wuhan 430030, China

**Keywords:** liver aging, innate immune cell, immunosenescence, inflammaging

## Abstract

Aging is a biological process with a gradual decline in functional capacity, and this process often enhances the risk of chronic disease morbidity and mortality. With advanced age, the immune system undergoes a process of remodeling that can lead to a chronic inflammatory state, termed immunosenescence and inflammaging, respectively. Immunosenescence is accompanied by changes in the number, proportion, and functional capacity of the innate immune cells. The accumulation of dysfunctional immune cells and the presence of low-grade inflammation can lead to organ damage and expedite the aging process. The liver, crucial in regulating the body’s metabolism and immune function, is not exempt from these effects. Age-related modifications affect its immune function and regenerative abilities, potentially increasing the prevalence of age-related liver diseases. While aging’s impact on the liver is relatively less severe compared to other organ systems, it still experiences an infiltration of innate immune cells and heightened inflammation levels. This review will elaborate on how aging affects the liver’s innate immune cells, such as neutrophils, macrophages, dendritic cells, mast cells, and innate lymphoid cells. It will also explore potential strategies for delaying immunosenescence to alleviate these age-related changes.

## 1. Introduction

In the elderly, there is a decline in the function of the immune system, also known as immunosenescence [1,2]. This results in a decreased ability to resist microorganisms’ infection and an increased risk of developing age-related diseases [3]. Moreover, immunosenescence has been recognized as detrimental due to its potential to contribute to a low-grade, persistent inflammation termed “inflammaging” [1]. Acute inflammation can protect the host against infection and repair tissue damage [4,5], but if the stressor persists, it will turn into chronic inflammation, leading to chronic inflammatory diseases. As individuals age, numerous factors lead to a rise in internally generated, displaced, or modified molecules produced by damaged or dead cells and organelles (cellular debris) [6], and these cellular remnants and constituents, including nucleic acids, mitochondrial DNA (mtDNA), cardiolipin, mitochondria, and other proteins, are referred to as damage-associated molecular patterns (DAMPs) [1]. Additionally, the capacity of proteasomes to degrade them through autophagy and/or mitophagy gradually declines with age [6]. The increased generation of DAMPs and impaired surveillance of damaged cells contribute to long-term inflammation activation, thereby further deteriorating with age.

The immune system, composed of immune cells, immune factors, and immune organs [7], undergoes significant adaptations during aging. Inflammaging mainly manifests as tissue degradation and changes in cytokine levels in the body [8]. Under various stimuli, immune cells can produce a variety of immune factors, which play an important role in inducing and sustaining inflammatory conditions. However, from an evolutionary perspective, it is argued that inflammaging is a process of immune system remodeling and adaptation due to chronic inflammation and inflammaging can be counteracted in the aging process by triggering a secondary adaptive response through the activation of anti-inflammatory networks [1]. Striking a balance between pro-inflammatory and anti-inflammatory responses is crucial for achieving healthy aging [9]. Furthermore, centenarians exhibit heightened anti-inflammatory capacities, indicating that the interaction between inflammation, immunity, and aging is likely to have a significant impact on the process of aging [10,11]. The involvement of immune cells is vital in both inflammaging and immunosenescence, and immune cells are also key targets in developing strategies to delay aging.

Polymorphonuclear cells (PMNs) and macrophages are the initial responders of the innate immune system in infection sites. They trigger an inflammatory reaction, ingest the pathogen, attract natural killer (NK) cells, and aid in the development and mitigation of dendritic cells (DCs) which connect the adaptive immune response [12]. The increasing DAMPs during aging can be recognized by the innate immune cell receptor and activate the adaptive immune response [6]. Immune cells can clear the senescence cells, but as individuals age, immune cells accumulate and their function declines [13]. It is well known that innate immunity plays a crucial role in the aging process, but the specific molecular mechanisms involved remain unclear. As the liver serves as a vital immune organ, it is paramount to investigate the changes in both the quantity and functionality of innate immune cells during liver aging, as well as their impact on liver aging.

**Figure 1:**
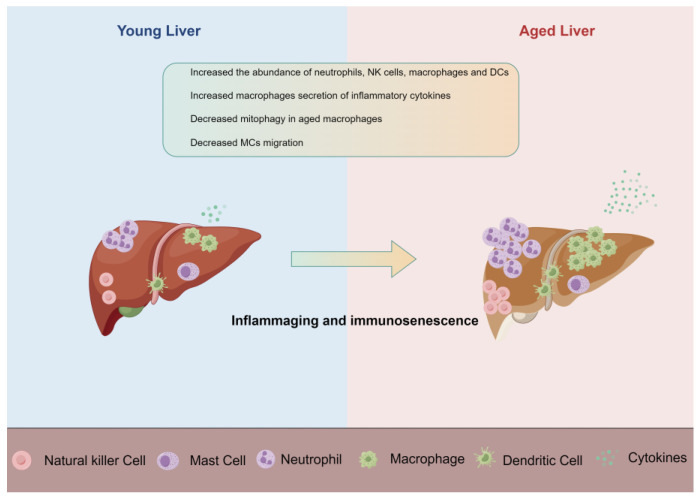
Innate immune cells change in the aging liver (by Figdraw 2.0). Schematic of changes in young and aged liver showing major changes in innate immune cells.

The liver, as an immunological organ, is irreplaceable in maintaining the balance of the immune system [14], which requires an intensely regulated response to continuous exposure to environmental challenges. The liver is supplied by both hepatic arteries and portal veins [14], and through the liver-gut axis, the liver holds the ideal position to detect and eliminate pathogens [15]. Hepatic immune homeostasis is maintained by a complex hepatic structure, as well as local and recruited immunocytes, which can clear pathogens and toxins while also regulating the immune response, including macrophages, NK cells, neutrophils, and so on [14]. Meanwhile, as an important metabolic organ [16], the liver can activate the immune system to counter the metabolic products to protect the liver itself and the entire body from metabolite-induced damage. However, the liver’s immune function declines during aging, and the abundance of innate immune cells in the aging liver also changes. Here, we review the changes in the different types of innate immune cells (Figure 1) during liver aging and their impact on liver aging. Additionally, we discuss strategies to target innate immune cells to ameliorate aging.

## 2. The Structure and Physiological Changes of Aging Liver

As individuals age, they are more susceptible to developing liver disease, while their liver’s ability to withstand damage decreases steadily [17]. The integrity of the liver structure contributes significantly to maintaining liver functions. The exchange of substances between liver cells and the bloodstream, as well as the communication between cells, contribute significantly to liver metabolism, detoxification, biosynthesis, and immune function [18]. The hepatic sinusoid is the most important structure for performing those functions. The liver receives the blood from the portal vein and hepatic artery and drains through the interlobular vein and the interlobular artery into the hepatic sinusoid. The hepatic sinusoid is vital for maintaining immune homeostasis in the liver [19]. Hepatic sinusoids, which are the capillary bed in the liver [20], are mainly composed of liver sinusoidal endothelial cells (LSECs), hepatic stellate cells (HSCs), and Kupffer cells (KCs) [18]. Especially, LSECs are deficient in the basement membrane, and the presence of diaphragmed fenestrae improves the crosstalk between cells [21]. The Disse space, which contains extracellular matrix components, separates the sinusoidal cells from the parenchymal cells [18]. The unique structure of the hepatic sinusoid provides a favorable site for intercellular communication, exchange of substances between blood and cells, and immune responses. However, the structure of the liver will change during aging.

The alteration of the LSECs and Disse during aging is called pseudocapillarization [22]. Age-related pseudocapillarization refers to the increasing thickness and decreasing number of fenestrations of LSECs, and endothelial markers change, such as up-regulation of the von Willebrand factor and intercellular adhesion molecule–1 (ICAM-1), along with the reduced expression of caveolin-1 [23]. Additionally, sporadic collagen and basal lamina deposit in the extracellular space of Disse [24]. In vivo, using quantitative liver intravital microscopy (qLim), reduced hepatic blood flow and increased sinusoidal vessel diameter were observed in the liver of aged mice [25]. Meanwhile, due to the decrease in hepatic blood flow, hepatic vascular resistance was increased in aged rats [18]. The studies have demonstrated a decline in liver mass and hepatic blood flow, a gradual loss of endothelial fenestration, as well as the activation of HSCs during the aging process [22,26]. Furthermore, qLim shows that inflammation was increased in the aged liver, due to the increase in proinflammatory macrophages, hepatic neutrophils, liver sinusoidal endothelial cells, senescent cells, and procoagulants [25].

## 3. Sequencing Technology Reveals the Cellular and Molecular Changes of Aging Liver

Additionally, there are also cellular and molecular changes in the aging liver. Advances in sequencing technology have allowed us to fully explore the cellular and molecular level changes during liver aging. The liver aging process is associated with an increase in inflammation-related genes and immune cell infiltration including macrophages, neutrophils, lymphocytes, and NK cells [27]. Moreover, single-cell transcriptomic analysis in mice revealed a reduction in the relative abundance of hepatocytes with age [28]. Simultaneously, differential gene expression analysis in aged liver highlighted an increased immune signature attributed to the overexpression of *H2-Aa*, *H2-Ab1*, *H2-D1*, *H2-Eb1*, *Cd74*, *Lyz2*, and other genes [28]. Single-cell analysis of aged mice demonstrated increased inflammatory activity in liver-resident macrophages, also known as Kupffer cells, including upregulation of the cytokine gene *IL1b*, contributing to hepatic inflammation and causing damage [28]. A sequencing in peripheral blood mononuclear cells (PBMCs) in young and old humans revealed that the relative percentage of NK cells and monocytes (MCs) was increased and the relative percentage of T cells (TCs), B cell (BCs) and DCs were decreased; additionally, the cell subtypes changed and shifted into effector and inflammatory cell populations [29].

Furthermore, the upregulation of liver aging biological pathways included tumor necrosis factor (TNF) signaling, interleukin-1 (IL-1) signaling, apoptotic signaling pathways, and the adaptive immune response [29]. In addition, sequencing results indicate that chromatin accessibility in the promoter regions was increased in an aging liver, while the transcriptional output maintained a relatively young-like state, due to a loss in the stability of the Pol II pausing complex, which is a ubiquitous step in the transcription cycle, upon aging [30]. In general, the sequencing results revealed enhanced immune signaling at the molecular and cellular levels in the liver during the aging process.

## 4. Innate Immune Cells

### 4.1. Neutrophil

Neutrophils, which play an important role in the innate immune response, have a range of immune functions, including phagocytosis, degranulation, reactive oxygen species (ROS) production, and releasing neutrophil extracellular traps (NETs) [31,32,33]. The web-like structures composed of chromatin, antimicrobial proteins, and enzymes released by neutrophils upon stimulation with IL-8 and phorbol 12-myristate 13-acetate (PMA) are called NETs [34]. Under physiological conditions, neutrophils are capable of clearing pathogens and preventing their dissemination, while also promoting tissue repair [35]. Also, neutrophils can act as detrimental players exacerbating inflammation. Mature neutrophils are found in two pools: a marginated pool that resides in certain tissues and a free-flowing intravascular blood pool [36]. Early studies suggested that the former pool in humans mainly resides in the liver, spleen, and bone marrow [37], and neutrophils can also be cleared from those tissues [38,39].

#### 4.1.1. Neutrophil Activation and Recruitment

**Figure 2:**
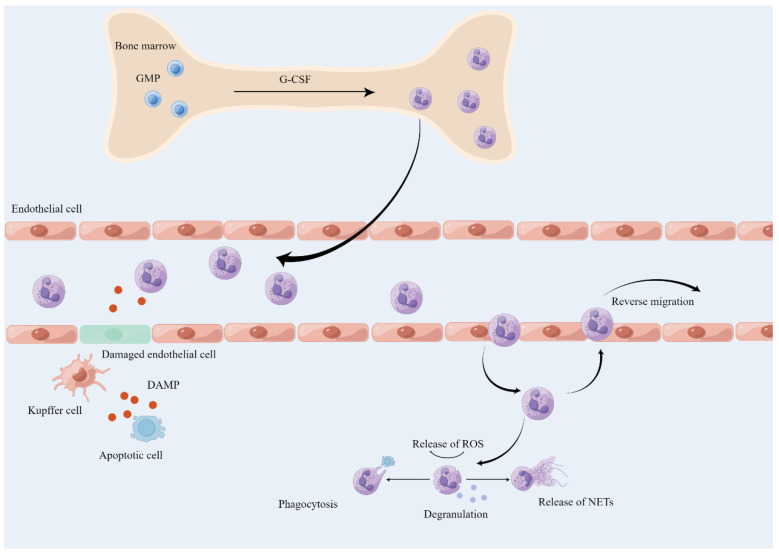
Overview of neutrophil recruitment and function in the liver (by Figdraw 2.0). During sterile inflammation, the product of G-CSF is increased and promotes the neutrophils released from the bone marrow. Neutrophils can sense the DAMP, activate the recruitment cascade, and migrate into the injury site through chemoattractant gradients. At the site of inflammation, neutrophils can exert their functions through degranulation, phagocytosis, production of ROS, and release of NETs. After completing their mission, neutrophils will be eliminated by macrophages or return to the bone marrow for disposal. GMP, granulo-monocytic progenitor.

Inflammation can be initiated by sterile stimuli, caused by tissue damage, or pathogenic origin. To maintain tissue homeostasis, one of the steps to counter inflammation is to recruit leukocytes, predominantly neutrophils [40]. The generation, maturation, and release of neutrophils all occur in the bone marrow, where the granulocyte colony-stimulating factor (G-CSF) promotes neutrophil development by binding to G-CSF receptors and facilitates their release by downregulating CXC-chemokine receptor (CXCR) 4 and upregulating CXCR2 in neutrophils [41] (Figure 2). Mature neutrophils are then released and respond to various stimuli such as chemokine (CXC-chemokine ligand (CXCL) 1, CXCL2) gradients, migrating to inflamed tissues [41]. Under sterile inflammation, DAMPs, such as extracellular ATP, are released from dead or damaged cells, and the sentinel cell KCs sense these signals, increasing the expression of IL-1β, which further actives ICAM-1 in LSECs and promotes the neutrophil adhesion [42]. Additionally, the adhesion of neutrophils with sinusoids depends on the integrin αMβ2 (Mac1) and its endothelial ligand ICAM-1 [42]. The chemokine CXCL2, released by active KCs, can lead neutrophils toward the inflammatory site between proximal 150 μm and 650 μm [42]. Furthermore, the neutrophil mitigation within the proximal 150 μm around the damage site needs the expression of formyl-peptide receptor 1 (FPR1) [42]. DAMPs from acetaminophen-induced hepatotoxicity in mice activate Toll-like receptor 9 (TLR9) on LSECs, which upregulate IL-1β and IL-18, as well as NACHT, LRR, and pyrin domain-containing protein 3 (Nalp3) inflammasome-mediated inflammation [43]. Neutrophil also can directly react to DAMPs by the TLR9/NF-κB pathway [44]. Platelets can aggregate in the sterile liver-afflicted area and promote neutrophil recruitment by the GPIIbIIIa-dependent mechanism [45]. The accumulation of proinflammatory CD11c+ macrophages in obese adipose tissue can secrete neutrophil chemotaxis proteins including CXCL14 and CXCL16 and increase hepatic neutrophil accumulation by the recruitment of neutrophils from the bone marrow [46]. Neutrophils can be recruited by the TLR2-S100A9-CXCL-2 pathway in liver inflammatory disease [47]. KCs can sense DAMPs and secret TNF-α, which actives the hepatocyte NF-κB pathway and promotes the neutrophil chemokine CXCL1 expression [48]. Therefore, KCs can sense the DAMPs and secret cytokines and chemokines to facilitate neutrophil homing, activation, adhesion, and accumulation in the liver.

Neutrophils have a short lifespan and can circulate in the blood or enter into damaged tissues, but ultimately, they are cleared. Aged neutrophils remaining in circulation upregulate CXCR4 and return to the bone marrow for clearance [49]. In addition, neutrophils in the site of acute inflammation are eliminated by macrophages [50]. And a study found that many neutrophils derived from acute sterile inflammation re-enter the vascular system and return to the lung for reprogramming and upregulate CXCR4 before entering the bone marrow, where they are eliminated by apoptosis [51].

The resolution of the inflammation can maintain homeostasis, if the stimulations are present for a long time, leading to chronic inflammation. Neutrophils are involved in the spontaneous resolution of liver inflammation and fibrosis via microRNA-223 [52]. The recruitment of neutrophils to the liver has a beneficial side, but its detrimental effects are predominant. Although there have been many studies on the liver and neutrophils, the relationship between neutrophils and liver aging remains relatively understudied.

#### 4.1.2. The Role of Neutrophil in Aged Liver

In aging individuals, neutrophil chemotaxis is impaired, and their antimicrobial capacity is reduced [53]. Additionally, decreased ROS production, diminished formation of NETs, and phagocytic dysfunction have been observed in aging individuals [53]. However, it has been reported that the number of neutrophils in peripheral blood increases with age in mice, and the formation of NETs is simpler during aging [54]. Neutrophils in aging rats exhibit a decline in phagocytic function [55]. However, the functional alterations of neutrophils in the aged liver have not been well studied, possibly due to the challenges associated with their acquisition. Senescent cells play a role in attracting neutrophils, whose pervasive presence in tissues is a key indicator of immunosenescence [56]. Increased neutrophil infiltration was reported in the liver during aging, due to the migration from the bone marrow, and decreased upon caloric restriction (CR) [57]. The number of Cxcl2+ macrophages increases in the liver of aged mice, and they can recruit neutrophils to the liver through the CXCL2-CXCR2 axis [58]. Neutrophils can induce paracrine senescence in the liver by ROS, inducing telomeric DNA damage [59]. During the aging process, the secretion of chemokines by activated HSC can promote excessive production of ROS in neutrophil infiltration in the niche, which leads to decreased activation and proliferation of liver stem cells in aging individuals, resulting in impaired liver regeneration [60]. In addition, it has been reported that CD4+ T cells require monocytes/macrophages to execute the killing of senescent hepatocytes, and selective depletion of neutrophils has marginally affected senescence surveillance [61], suggesting that neutrophils might not directly clear senescence cells.

Interestingly, it is reported that senescence endothelial cells, in retinopathy, can secrete cytokines to recruit neutrophils and provoke the release of NETs, and NETs eliminate senescence endothelial cells for remodeling retinal vasculature through promoting apoptosis [62], which arouses the concern about aging and NETs. However, it has been shown that histones and high mobility group box-1 (HMGB1), acting as DAMPS, induce NET formation, and NETs are cytotoxic to hepatocytes and pro-inflammation during liver ischemia/reperfusion (I/R) injury [63]. In addition, neutrophil elastase, the component of NETs, can contribute to hepatocyte apoptosis through the activation of caspase-3 in rats [64]. Therefore, the relationship of neutrophils or NETs on aging liver deserves future investigations.

Neutrophil accumulation in the aging liver has been confirmed [57], though the underlying molecular mechanisms remain elusive. Possible explanations for this phenomenon include increased neutrophil release from the bone marrow and chemokine production by macrophages. The observed decrease in neutrophil infiltration when liver aging is delayed by caloric restriction (CR) hints at the role neutrophils may play in this process [57]. However, the interplay between chronic low-level sterile inflammation and neutrophil presence during liver aging warrants further study. We have thoroughly delineated the journey of neutrophils from their origin in the bone marrow to their recruitment and eventual clearance in the liver under sterile inflammation conditions, providing a solid framework for future research into neutrophil infiltration associated with liver aging. It is plausible that liver aging could foster neutrophil infiltration by disrupting a specific pathway involved in their lifecycle.

### 4.2. Macrophage

Macrophages exist in two phenotypes, the pro-inflammatory M1 phenotype and the anti-inflammatory/matrix remodeling M2 phenotype, and the transition of macrophage phenotypes between these two polarizations depends on signals from the extracellular environment [65]. M1 polarization of macrophages can be induced by lipopolysaccharides (LPS) and type 1 cytokines (such as IFN-γ), and it is related to pro-inflammatory responses [66], while M2 polarization is triggered by type 2 cytokines (such as IL-4 and IL-13), and it is associated with counteracting inflammatory responses and modulation of tissue repair [67]. The polarization of macrophages is highly complex, and the functions of M1 and M2 types may not always correspond directly with their functions in different scenarios [19,68], for example, under conditions of aging, the number of M2-type macrophages increases, but they primarily play a pro-inflammatory role [68]. Therefore, we must dynamically understand the polarization of macrophages and their characteristics.

Aging has been shown to influence the phenotype and function of macrophages, although its precise effects at the cellular and molecular levels remain poorly understood. With increasing age, there is an increase in the basal expression of transglutaminase-2, a conserved M2 marker that is observed in both mice and humans [69,70]. Therefore, M2-like macrophages appear to accumulate with age. TLRs are also a component of pattern recognition receptors (PRRs), capable of responding to pathogen-associated molecular patterns (PAMPs) and DAMPs. However, during the aging process, the expression of TLRs on macrophages decreases [3]. The decline of TLRs during the aging process may impact the wound repair capacity of macrophages through their crucial role in the programming of macrophage cytokine secretion and other angiogenesis factors by an interaction with the adenosine A2a receptor [3]. In aging mice, there is a significant reduction in respiratory burst activity and phagocytic capacity of macrophages, as well as increased oxidative stress, indicating impaired functionality of KCs during the aging process [71]. The impaired resolution of acute inflammation in the elderly, characterized by decreased T-cell immunoglobulin mucin receptor 4 (TIM-4) expression on macrophages and reduced efferocytosis that can clean apoptotic bodies, can be alleviated by administering a P38 inhibitor to enhance apoptotic neutrophil clearance [72]. Therefore, during the aging process, the ability of macrophages to defeat pathogenic microorganisms, phagocytic capacity, ability to cope with various oxidative stresses, cytokines secretion, and tissue repair capabilities are affected.

#### 4.2.1. Macrophage in the Liver

Macrophages can be divided into tissue-resident macrophages and monocyte-derived macrophages (MoMϕs) [65]. KCs are the most abundant immune cells in the liver [73]. KCs are a component of the hepatic reticuloendothelial system, which is also known as the mononuclear phagocyte system, and they work together with other immune cells to combat pathogens [19]. The surface markers of murine hepatic macrophages differ, with MoMϕs expressing CD11b^+^, F4/80 ^intermediate (int)^, Ly6C+, and CSF1R+, while KCs express CD11b^low^, F4/80^high^, and cle4f^+^ [74]. In addition, in the human liver, Kupffer cells are composed of CD68^+^MARCO^+^ cells, CD68^+^MARCO^−^ macrophages, and CD14^+^ monocytes [75,76]. KCs derive from CSF1R+ erythromyeloid progenitors (EMPs) of yolk sac origin, which are present in the fetal liver during embryonic development [77]. On the other hand, the MoMϕs are from bone marrow-resident hematopoietic stem cells [19,74].

Macrophages, as sentinel cells, play an important role in maintaining liver stability. The main functions of KCs include clearing cellular debris and metabolic waste, maintaining iron homeostasis by phagocytosing red blood cells and subsequently iron recycling, regulating cholesterol homeostasis by producing cholesterol ester transfer protein, resisting pathogenic infection, and promoting immune tolerance [75]. When liver damage occurs, KCs are the first to respond to the disruption, interact with other hepatic cells, and release chemokines to recruit the leukocytes, and the recruited monocytes differentiate into MoMϕs [75].

#### 4.2.2. Macrophage in the Aging Liver

It is well established that the number of macrophages was increased in the aged liver [18,78]. In the liver of aged mice, there is an increase in CD38+ KCs that exhibit pro-inflammatory M1-like characteristics [79]. P16-Cre knockin mice crossed with a Rosa26-mTmG reporter can continuously label p16-expressing cells, and it was found that p16^High^ cells appear in large quantities in the liver with age [80]. Further research has shown that these cells are mainly vascular endothelial cells and F4/80-positive macrophages [80]. Aging inhibits mitophagy in aged macrophages by mediating a decrease in PTEN-induced kinase 1 (PINK1)/Parkin-mediated mitochondrial ubiquitination and impairing lysosomal biogenesis and function through the regulation of mTOR/transcription factor EB (TFEB) signaling [81]. As a result, this promotes macrophage-mediated leakage of mtDNA into the cytosol and subsequent activation of STING, contributing to the development of sterile inflammation in the liver [81]. Macrophages phagocytosed miR-30b-5p-containing extracellular vesicles derived from senescent cells, leading to a decrease in their SIRT1 expression; this, in turn, facilitated the entry of p65 into the nucleus, activating canonical NF-κB signaling, promoting cytokine production in macrophages, and the study also found that miR-30b-5p increased with aging in the liver [82]. Indeed, there is reason to speculate that the accumulation of macrophages during the aging process in the liver further promotes liver aging and the development of age-related diseases.

After partial hepatectomy in old mice, neuropilin-1 (NRP1)-dependent suppression of endothelial protein C receptor (EPCR) activates platelets and macrophages to format the platelet-macrophage rosette, promoting fibrosis rather than regeneration [83]. In an acute liver injury mouse model, hepatocyte senescence transmission is dependent upon transforming growth factor-beta (TGFβ) derived from macrophages [84]. Hepatic macrophages are increased in the aged liver and secrete pro-inflammatory cytokines, particularly IL-6, which contribute to age-related pathologies [85].

Above all, the senescence-associated secretory phenotype (SASP) secreted by senescent cells can induce chronic inflammation, which in turn can lead to tissue damage and dysfunction. The presence of macrophages in the liver can exacerbate this process, as they are an important source of pro-inflammatory cytokines and can also promote the infiltration of other immune cells. Furthermore, macrophages can directly contribute to tissue damage through the production of reactive oxygen species and other detrimental molecules. All these factors can ultimately lead to the development of liver diseases such as non-alcoholic fatty liver disease, fibrosis, and cirrhosis.

### 4.3. Other Innate Immune Cells

The role of other innate immune cells in liver aging is not well-studied in comparison to neutrophils and macrophages, thus we will not delve into this topic extensively.

#### 4.3.1. Dendritic Cells in Liver Aging

DCs are specialized antigen-presenting cells that play a crucial role in immune surveillance by presenting antigen peptides to lymphocytes [13]. Murine liver DCs can be divided into two types: classical or conventional DCs (cDCs) and plasmacytoid DCs (pDCs) [86,87]. Liver DCs are mainly located in the perivenular region, portal space, and beneath the Glisson’s capsule, whereas a small number of DCs are also scattered throughout the parenchyma of the liver [88]. In humans, BDCA-1 (+) DCs are the most common liver DC subset, while most peripheral blood DCs are CD16 (+) [89]. DCs can induce liver tolerance, as human liver DCs are believed to promote immune hypo-responsiveness [89]. Infusing donor-derived regulatory dendritic cells before liver transplantation has been found to reduce the frequency of CD8+ T cells and NK cells, as well as TH1 pro-inflammatory cytokines, after 12 months [90]. Aging is associated with an increase in dendritic cell (DC) numbers in the liver, and there is an age-related change in subpopulations, including increased pDCs and CD4-CD8α+ DCs in the liver [91]. Furthermore, long-term administration of Lactococcus lactis-strain plasma can maintain the immune system by activating pDCs, thereby slowing down aging and extending lifespan [92].

#### 4.3.2. Mast Cell in Liver Aging

It is known that the density of mast cells (MCs) increases during aging [93,94]. Compared with untreated aging rats and aging rats intraperitoneally injected with carbon tetrachloride CCL4, young rats exhibited a stronger recruitment ability and faster recruitment speed of MCs in the liver compared to aged rats [95]. Furthermore, in multi-drug resistant gene 2 (Mdr2−/−) mice, treatment with vivo-morpholino resulted in downregulation of the liver stem cell factor (SCF), a chemoattractant for c-kit expressed on MCs, thereby inhibiting mast cell migration and reducing biliary injury/senescence and liver fibrosis [96].

#### 4.3.3. Innate Lymphoid Cells in Liver Aging

The innate lymphoid cell (ILC) family consists of NK cells and ILCs which are composed of ILC1s, ILC2s, and ILC3s [97,98]. The number of NKs in PBMC is increased in Lewis rats during aging [99]. The research demonstrated that the ability of NK cells to exert antiviral activity decreases as inflammatory states are upregulated during the aging process [29]. In addition, the number of NKs increased in the aging mice liver [27]. The liver of aged mice exhibited a decrease in CD49b^−^CD49a^+^ tissue-resident NK cells, an increase in CD49b^−^CD49a^−^ NK cells, and an overall decrease in the number of CD49b^−^ NK cells [100]. Additionally, NK cells aid in removing senescent cells during liver fibrosis and reduce fibrosis progression [101]. The research demonstrates that NK cells target and eliminate senescent cells through granule exocytosis, playing a protective role against liver fibrosis [102].

In addition, ILC1s is the most abundant type of ILC in the liver [103], and the number of ILC1s in the liver decreases during aging [104]. The research on ILCs in liver aging is still limited, but ILCs play a dual role in liver aging-related diseases. Inhibiting the expression of HSP70 blocks the secretion of IL-13 in ILC2 cells, leading to the suppression of hepatocellular carcinoma (HCC) progression [105]. Through the induction of CXCL2, ILC2s facilitate the progression of HCC by promoting immunosuppression mediated by neutrophils [106].

## 5. Strategies of Ameliorating Aging-Related Immunosenescence

Aging affects the immune system and decreases the ability to prevent infection, and immune cell accumulation in special aging tissues can promote the process of aging. Nevertheless, aging is a malleable process that can be slowed down by implementing strategies like proper nutrition and medicinal interventions [107,108]. Similarly, the aging of the immune system is also plastic, and by enhancing the activation of the innate immune system, “healthspan”, a longer period of good health, can be achieved [109].

### 5.1. Senolytic Therapy

Under normal conditions, aging cells can influence their surrounding environment through the secretion of SASP, including extracellular matrix-degrading enzymes, cytokines, chemokines, immune modulatory factors, proteases, hemostatic factors, ceramides, bradykinins, and DAMPs [110,111,112]. SASP can recruit immune cells, which in turn clear senescent cells [113]. However, the ability of immune cells to eliminate senescent cells decreases during aging. Moreover, senescent cell accumulation occurs continuously during liver aging [114]. Therefore, it is necessary to develop strategies for clearing senescent cells.

The drugs that selectively kill senescent cell are called “senolytic” [112]. The combination of the anti-aging drugs dasatinib and quercetin (D+Q) can reduce hepatic lipid degeneration and steatosis by targeting the accumulation of senescent cells [115]. By inhibiting thrombomodulin (THBD) signaling, a key pathway in the fate of senescent cells by vorapaxar, it is possible to eliminate senescent cells during the hepatic fibrosis process and maintain liver homeostasis [116]. Moreover, using the vorapaxar also can ameliorate nonalcoholic fatty liver disease progression by targeting the senescent hepatocytes [117]. Furthermore, chimeric antigen receptor (CAR)T cells targeting the urokinase-type plasminogen activator receptor (uPAR) can serve as senolytic drugs to eliminate senescent cells and reduce CCl4-induced or nonalcoholic steatohepatitis-induced liver fibrosis [118]. However, promoting cellular senescence can also play a beneficial role in age-related liver diseases. The induction of senescence by TGFβ in hepatocellular carcinoma cells leads to the inhibition of tumor growth [119]. Activation of the p53 in mouse hepatocellular carcinoma induces cellular senescence, ultimately promoting tumor regression [120]. Overall, the benefits of clearing accumulated senescent cells outweigh the drawbacks.

### 5.2. Caloric Restriction

CR refers to a long-term reduction in total calorie intake without causing malnutrition [121] and is considered one of the effective interventions for delaying aging [122]. In aged liver tissue, age-related β-galactosidase staining increases, and CR can reverse this phenomenon [57]. Autophagy has strong anti-aging activity, and caloric restriction can reverse the changes in autophagy-related genes caused by liver aging [123]. In addition, CR can alter the disrupted ecosystem of the number and proportion of immune cells [57]. CR can prevent age-related decreases in citrate synthase activity, protein levels of TFAM, MFN2, DRP1, and mtDNA content in the liver of 28-month-old rats [124]. Metformin [125] and resveratrol [126] are common CR mimetics that can imitate the effects of CR through pharmacological intervention. Metformin effectively retards the progression of hepatic cyst formation and fibrosis in polycystic kidney (PCK) rats by activating AMP-activated protein kinase (AMPK) and inhibiting the signaling pathways that contribute to cellular proliferation and fibrotic processes in the liver [127]. Metformin promotes communication between KCs and hepatocytes, regulating lipophagy and necroptosis processes, and ultimately reducing hepatic fat accumulation in cases of hepatic steatosis [128]. Resveratrol has been shown to reduce inflammation in the liver of aged mice [129].

### 5.3. Exercise

Regular physical exercise has been widely recognized as a way to increase longevity, and recent evidence suggests that it can also promote a healthier immune system by reducing inflammation and enhancing immune-cell functionality. Regular exercise can delay the onset of immunosenescence and alleviate the inflammaging [130,131]. Moreover, moderate exercise can rescue the decline of age-related neutrophil phagocytic function [132]. Resistance training over an extended period has the potential to enhance NK cell cytotoxicity and mitigate the decline in immune function that is associated with aging in postmenopausal women [133]. Elderly women who are physically fit demonstrated significantly better NK cell function compared to inactive individuals [134]. Exercise can mitigate the infiltration of CD45+ immune cells and neutrophils in the liver of aging mice and decrease the extent of inflammation in this organ [135]. In addition, exercise can reverse the expression of IL-1β, P21, and the accumulation of lipid droplets in the liver of aging mice [135].

### 5.4. Cell Transplantation

Young immune cells can alleviate the detrimental effects of aging immune cells during the aging process. Transplanting young immune cells into excision repair cross-complementing group 1 (Ercc1) knockout mice, which selectively increases the burden of endogenous DNA damage and induces aging primarily in the immune system, alleviates the aging process in ERCC1 defective mice [136]. In addition, when recipient mice were given splenocytes from aged mice, several non-lymphoid organs showed increased levels of aging markers like p16 and p21 compared to those from young mice [136]. This indicates that aging immune cells can drive overall aging in the body, and the transplantation of young immune cells can delay aging. The separate administration of NK cells or the combined administration of NK cells and Acein, a dopamine-releasing peptide, in mice, can reduce the expression of liver aging markers P16 and P21, as well as the SASP level, and increase the senescent cells clearance [137]. Centenarians maintain some efficient immune responses in NK cells, which may play a crucial role in their successful aging [138]. Research has shown that transferring macrophages isolated from the livers of young mice to old mice can alleviate the severity of ischemia-reperfusion injury (IRI) in old mice [58]. On the other hand, transferring macrophages from old mice to young mice worsens the extent of IRI in the mice [58]. Therefore, the key to anti-aging and longevity is to maintain the quantity and activity of immune cells in the body through cell transplantation. However, despite the advances in cell transplantation, there are still numerous hurdles to overcome in its clinical application due to the heterogeneity of the human body and the intricate cellular makeup.

## 6. Conclusions and Perspectives

Aging poses a significant risk for liver-related diseases. As the liver serves as a vital immune organ, the maintenance of liver stability relies heavily on the interactions and functionality of innate immune cells. However, during the aging process, the composition of these immune cells undergoes changes that contribute to liver aging. To develop effective strategies for preventing and slowing down aging, it is imperative to gain a deeper understanding of the mechanisms and alterations that occur during this process.

Recent advancements in single-cell sequencing technology and multi-omics approaches have provided a more comprehensive understanding of the quantitative, proportional, phenotypic, and functional changes in specific cell types during aging. Nevertheless, the reasons behind the changes in the number and subsets of liver resident innate immune cells, as well as their underlying functions in liver aging, remain unclear. Moreover, there is a need for further investigation into the impact of interactions between innate immune cells on liver aging.

In the future, with continued technological development, the role of immune cells in the aging liver will be further explored. This will ultimately contribute to a better understanding of liver aging and facilitate the development of targeted interventions to mitigate its effects.
